# Strategies for coping with stress in early adolescents and their association with sociodemographic risk factors

**DOI:** 10.1192/j.eurpsy.2025.1142

**Published:** 2025-08-26

**Authors:** J. Hamidovic, L. Dostovic Hamidovic, A. Brigic, M. Mesanovic

**Affiliations:** 1 Department of Psychiatry; 2 Department of Pediatrics, University Clinical Center Tuzla, Tuzla, Bosnia and Herzegovina

## Abstract

**Introduction:**

The concept of dealing with stress refers to different forms of behavior that an individual uses in response to stressful situations, with the aim of reducing the effects of those stressful events or situations. Ways of coping are important as a prerequisite for positive growth and development and can be a significant factor in the etiology of psychological and somatic problems. Coping strategies include all those behaviors that are used to overcome difficulties in stressful situations. They refer to emotional, cognitive and behavioral responses to stressful situations that vary with time and characteristics of a specific stressful situation. In adolescence, the child encounters various forms of life situations and stresses, without yet having developed a wide range of coping strategies on which to rely. Children’s growth and development and the influence of sociodemographic factors represent certain risks that can directly or indirectly affect stress coping strategies.

**Objectives:**

The aim is to determine ways of coping with stress in early adolescents and their connection with sociodemographic risk factors.

**Methods:**

We analyzed a group of 240 early adolescents (11-15 years) from Tuzla Canton, Bosnia and Herzegovina, in the general population. The sample was chosen because the early adolescent period is vulnerable in children’s growing up and psychological development. The Kidcope coping scale for children and adolescents was used to assess the way of coping. The data were processed using the method of descriptive statistics. Pearson’s correlation test was used to assess the association between sociodemographic risk factors and coping methods.

**Results:**

On the coping scale, early adolescents showed a statistically significantly higher level of self-blame and blaming others. On the questionnaire of psychosomatic symptoms (PS), the obtained results show that living in a rural area leads to frequent blaming of others as a way of coping (p<0.05), as well as a significantly higher level of prayer (p<0.05). Higher economic status and higher monthly income are significantly positively associated with resignation (p<0.01). Higher educational status of the mother and better living conditions are also positively related to resignation as a way of coping with stress (p<0.05). Disturbed family relationships are associated with a higher frequency of distraction (p<0.05).

**Conclusions:**

Early adolescents show significant levels of various stress coping strategies. There is a significant correlation between sociodemographic risk factors (place of residence, economic status of the family, disturbed family relations) and psychosomatic symptoms in early adolescents.

**Disclosure of Interest:**

None Declared

